# Accuracy of self-reported height, weight, and waist circumference in a general adult Chinese population

**DOI:** 10.1186/s12963-016-0099-8

**Published:** 2016-08-11

**Authors:** Shurong Lu, Jian Su, Quanyong Xiang, Jinyi Zhou, Ming Wu

**Affiliations:** Department of Chronic Disease, Jiangsu Provincial Center for Disease Control and Prevention, 172, Jiangsu Road, 210009 Nanjing, China

**Keywords:** Accuracy, Adults, Body height, Body mass index (BMI), Body weight, Obesity, Waist circumference (WC)

## Abstract

**Background:**

Self-reported height, weight, and waist circumference (WC) are widely used to estimate the prevalence of obesity, which has been increasing rapidly in China, but there is limited evidence for the accuracy of self-reported data and the determinants of self-report bias among the general adult Chinese population.

**Methods:**

Using a multi-stage cluster sampling method, 8399 residents aged 18 or above were interviewed in the Jiangsu Province of China. Information on self-reported height, weight, and WC, together with information on demographic factors and lifestyle behaviors, were collected through structured face-to-face interviews. Anthropometrics were measured by trained staff according to a standard protocol.

**Results:**

Self-reported height was overreported by a mean of 1.1 cm (95 % confidence interval [CI]: 1.0 to 1.2). Self-reported weight, body mass index (BMI), and WC were underreported by −0.1 kg (95 % CI: −0.2 to 0.0), −0.4 kg/m^2^ (95 % CI: −0.5 to −0.3) and −1.5 cm (95 % CI: −1.7 to −1.3) respectively. Sex, age group, location, education, weight status, fruit/vegetable intake, and smoking significantly affected the extent of self-report bias. According to the self-reported data, 25.5 % of obese people were misclassified into lower BMI categories and 8.7 % of people with elevated WC were misclassified as normal. Besides the accuracy, the distribution of BMI and WC and their cut-off point standards for obesity of a population affected the proportion of obesity misclassification.

**Conclusion:**

Amongst a general population of Chinese adults, there was rather high proportion of obesity misclassification using self-reported weight, height, and WC data. Self-reported anthropometrics are biased and misleading. Objective measurements are recommended.

## Background

As the most commonly used measure of obesity in adults, body mass index (BMI, kg/m^2^) is calculated as weight (kg) divided by squared height (meters). Knowledge on the weight and height of a population enables subgroups to be identified that are at increased risk of developing obesity-related health problems and dying prematurely [1, 2]. As self-reported height and weight are easier, cheaper, and quicker to collect compared with direct measurements [3], they have been widely used in disease surveillance, web-based studies, telephone interviews, and other types of research [4, 5]. Previous studies suggest that, although self-reported data on height and weight were positively correlated with measured data [5], adults tend to overreport height and underreport weight, especially women, elderly people, or those with higher weight [6–8]. Inaccuracies in self-reported height and weight lead to biased calculations of BMI, and consequently to inaccurate assessment of the disease and mortality risk of a population [2, 6, 9].

While BMI is a measure of overall obesity, waist circumference (WC) is an internationally used measure of abdominal obesity [1]. In the limited number of studies that have been performed on the accuracy of self-reported WC in adults, underreporting of WC has been the most consistent finding [2, 8, 10]. Very few studies assessed the accuracy of self-reported WC as well as that of self-reported weight, height, and BMI in the same population [2, 10].

Some studies have investigated the association between the accuracy of self-report bias and certain sociodemographic characteristics besides sex, age, and BMI status [2, 7, 9, 11], such as socioeconomic status [2, 11] and ethnicity [7, 9, 11]. Low socioeconomic status was consistently related to greater self-report bias, but evidence for ethnicity is inconsistent. A limited number of studies examined the effect of certain lifestyle factors on the accuracy of self-reported anthropometrics. For example, smoking [2, 12] was found to be significantly related to reduced bias. Studies suggested that diet may be another behavioral factor associated with the accuracy of self-reported data [10]. However, no association between the accuracy of self-reported anthropometrics and alcohol consumption or physical activity level has been identified, despite both being important behavioral risk factors for obesity.

Instead of targeting a general population, most studies on accuracy or validity of self-reported data focused on subgroup populations, such as overweight population, middle-aged adults, or college students [2, 5, 8, 10]. The limited number of studies performed at a general population level, mostly in Western countries [13, 14], have revealed substantial differences in self-reported anthropometrics bias between populations of different countries [13]. The accuracy and determinants of bias in self-reported anthropometrics of general adults of China have not been studied yet. China differs from Western countries both in the epidemical characteristics of obesity and in social desirability toward obesity. On the one hand, even with a substantial increase from 7.1 % in 2002 to 12.0 % in 2010 [15, 16], the prevalence of obesity is still relatively low compared to that of Western countries. On the other hand, because of earlier experiences with long-term poverty and famine, the Chinese traditionally believe that being overweight is a sign of happiness and abundance rather than a health problem [17]. Therefore, we believe that this country-specific study, which aimed to examine the accuracy of self-reported height, weight, and WC, is needed and will enrich the literature of self-reported anthropometrics in non-Western countries.

## Methods

### Setting and participants

The data presented in this article are from the *2013 Jiangsu Provincial Surveillance Survey on Chronic Disease and Behavioral Risk Factors*, a community-based cross-sectional survey, ethically approved by the Institutional Review Board of the Jiangsu Provincial Department of Health. Jiangsu Province lies in the southeast of China, with a population of 79.6 million. There are 13 cities in Jiangsu that comprise 98 counties/districts (representing rural areas and urban areas, respectively, according to the Chinese administrative division criteria), of which 14 are provincial disease surveillance points (DSPs). These 14 DSPs are representative of Jiangsu province in terms of geographical distribution, economic development, and population composition [18].

A multi-stage cluster sampling method was employed to select participants. Firstly, four towns/streets were selected from each DSP by proportion to population size sampling method [19]. Secondly, three villages/communities were selected from each selected town/street. Within each selected village/community, 50 households (private dwellings) were randomly sampled. By the Kish Grid method [20], one eligible resident (aged 18 or more, residing in the household for a minimum of six months prior to the survey) was selected to participate in the survey from all eligible people within each household. The target sample size was 600 respondents per DSP and 8400 (600/DSP*14DSPs) for the total.

### Procedure

Local village/community general practitioners invited the selected residents to participate in the study and informed them of the specific location and time. All participants gave written informed consent upon arriving at the survey site, which were primarily village/community health service stations.

Each participant completed a face-to-face structured interview performed by a trained public health worker. The survey inquired about demographic characteristics and lifestyle factors (including smoking, alcohol drinking, diet, and physical activity), and asked *Do you know your current height/weight/WC*?. If participants reported knowing their current height, weight, and/or WC, they were asked to give the specific values (referred to as “self-reported data” in this analysis). “Current daily smoking” was defined as smoking at least once a day and “drinking alcohol within 30 days” was defined as having any alcoholic drink within the past 30 days. Information on the intake of meat and fruits/vegetables were collected by food frequency questionnaire. Furthermore, physical activity level was categorized according to the Global Physical Activity Questionnaire Analysis Guide of the World Health Organization [21]. Details of the interview and the methods for categorizing lifestyle factors have been described elsewhere [22].

Direct measurements of height, weight, and WC were taken by a team with two trained staff. The reliability and validity of the measurements of each team were tested in a standardized method by a local supervisor during staff training [22]. According to a standard protocol, height was measured to the nearest 0.1 cm without shoes using a stadiometer, weight was measured without shoes and excess clothing to the nearest 0.1 kg using digital scales, and WC was measured halfway between the inferior margin of the last rib and the iliac crest in the mid-axillary plane to the nearest 0.1 cm with a waist circumference measuring tape. For WC, if the variation between the first and second measurements was greater than 2 cm, a third measurement was taken and the mean of the two closest measurements was calculated. These data are referred to as directly measured (DM) for the purpose of this study. All anthropometric measurements were completed in the morning. For weight and WC measurements, eight to 10 h of fasting was required.

### Statistical analysis

BMI was calculated based on self-reported and measured height and weight. The BMI categories were identified using Chinese cut-off points as underweight (<18.5 kg/m^2^), normal (18.5–23.9 kg/m^2^), overweight (24.0–27.9 kg/m^2^), and obese (≥28.0 kg/m^2^) [23], and using the World Health Organization standard as underweight (<18.5 kg/m^2^), normal (18.5–24.9 kg/m^2^), overweight (25.0–29.9 kg/m^2^), and obese (≥30.0 kg/m^2^) [1]. WC was grouped into “elevated” (men ≥ 90 cm/women ≥ 80 cm or men ≥ 102 cm/women ≥ 88 cm) and “normal” (men < 90 cm/women < 80 cm or men < 102 cm/women < 88 cm) according to the International Diabetes Federation standard for the Chinese or for the Americans [24]. Reliability between self-reported and measured values of continuous variables was evaluated with the use of intraclass correlation and 95 % confidence intervals. Bland-Altman plots were used in order to examine the individual agreement between self-reported and measured anthropometrics [25]. Group comparisons were performed using analysis of variance or *χ*^2^ tests as appropriate. Blocks of variables (sociodemographic factors, weight status, and lifestyle behaviors) were entered into the hierarchical regression models to allow analysis of their contributions to self-report bias after controlling for previously entered variables.

Data management and statistical analyses were performed using SPSS (V22.0, Chicago, Illinois) and *P* < 0.05 (two sided) was considered to be significant.

## Results

**Fig. 1:**
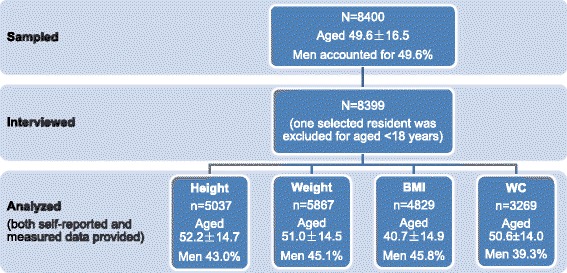
Response rate and sample size of analysis. BMI: body mass index; WC: waist circumference

As shown in Fig. 1, there was a total of 8399 residents from the sample of 8400 who were interviewed, aged 18.0 to 93.7 years (average, 52.2 ± 14.7 years), and almost half were males (49.6 %). Participants with self-reported and measured height, weight, and WC were included in the analyses.

The intraclass correlation coefficients between self-reported and measured was 0.957 (95 % CI: 0.955, 0.960) for height, 0.959 (95 % CI: 0.957, 0.961) for weight, 0.924 (95 % CI: 0.920, 0.928) for BMI, and 0.918 (95 % CI: 0.912, 0.923) for WC, respectively, which demonstrated very high concordance between self-reported and measured data.

Descriptive statistics on the difference between self-reported and measured height, weight, BMI, and WC by sociodemographic characteristics were shown in Table 1. The mean difference (self-reported minus measured) was 1.1 cm (95 % CI: 1.0, 1.2) for height, −0.1 kg (95 % CI: −0.2, 0.0) for weight, −0.4 kg/m^2^ (95 % CI: −0.5, −0.3) for BMI, and −1.5 cm (95 % CI: −1.7, −1.3) for WC, respectively. Compared with men, women showed much greater error in self-reported WC (−1.9 vs. − 0.9, *P* < 0.001). The difference between self-reported and measured height increased significantly with increasing age (*P* < 0.001). The difference between self-reported and measured weight, BMI, and WC were statistically significant among age groups as well, all *P* < 0.05. Geographical location and level of education were related to a significant difference between self-reported and directly measured weight and WC, and household income level was significantly related to that of weight, all *P* < 0.05.

**Table 1:** The difference between self-reported and measured (SR-DM) height, weight, BMI, and WC by sociodemographic factors in Jiangsu, China

		Height (cm)			Weight (kg)			BMI (kg/m^2^)			WC (cm)		
		n	Mean	95 % CI	n	Mean	95 % CI	n	Mean	95 % CI	n	Mean	95 % CI
Total		5037	1.1	1.0,1.2	5867	−0.1	−0.2,0.0	4829	−0.4	−0.5,-0.3	3269	−1.5	−1.7,-1.3
Sex	Men	2377	1.1	0.9,1.2	2645	−0.1	−0.2,0.1	2278	−0.4	−0.4,-0.3	1285	−0.9	−1.2,-0.5
	Women	2660	1.2	1.1,1.3	3222	−0.1	−0.2,0.1	2551	−0.4	−0.5,−0.4	1984	−1.9	−2.1,−1.7
	*P* value	0.098			0.882			0.13			<0.001		
Age group (years)	18–29	569	0.5	0.4,0.7	594	−0.4	−0.8,0.1	534	−0.3	−0.5,−0.1	292	−1.2	−1.8,−0.6
	30–39	661	0.7	0.5,0.9	704	−0.6	−0.8,−0.3	637	−0.4	−0.5,−0.3	426	−1.9	−2.4,−1.5
	40–49	1221	0.9	0.8,1.0	1389	−0.1	−0.3,0.2	1169	−0.3	−0.4,−0.2	837	−1.9	−2.3,−1.5
	50–59	1271	1.2	0.9,1.4	1512	0.0	−0.2,0.1	1224	−0.4	−0.5,−0.3	851	−1.2	−1.5,−0.9
	60–69	893	1.6	1.4,1.8	1134	0.2	0.0,0.4	863	−0.5	−0.5,−0.4	598	−1.4	−1.9,−0.9
	70~	422	2.3	1.9,2.7	534	0.3	−0.2,0.7	402	−0.6	−0.9,−0.4	265	−1.0	−1.6,−0.3
	*P* value	< 0.001			0.002			0.025			0.015		
Locations	Urban	3982	1.2	1.1,1.3	4223	−0.2	−0.3,−0.1	3875	−0.4	−0.5,−0.4	2592	−1.3	−1.5,−1.1
	Rural	1055	1.0	0.8,1.2	1644	0.3	0.0,0.5	954	−0.2	−0.4,−0.1	677	−2.2	−2.7,−1.7
	*P* value	0.078			< 0.001			0.003			0.001		
Education	Primary	1495	1.4	1.2,1.7	2107	0.2	0.0,0.4	1392	−0.4	−0.5,−0.4	1027	−1.7	−2.1,−1.4
	Secondary	2994	1.1	1.0,1.2	3218	−0.1	−0.3,0.0	2902	−0.4	−0.5,−0.3	1927	−1.5	−1.7,−1.2
	University	548	0.6	0.4,0.8	542	−0.7	−1.0,−0.4	535	−0.4	−0.5,−0.3	315	−0.9	−1.4,−0.4
	*P* value	0.347			< 0.001			0.545			0.037		
Household income level	Low	1034	1.3	1.1,1.4	1392	0.3	0.0,0.6	962	−0.3	−0.5,−0.2	652	−1.6	−2.0,−1.1
	Moderate	2550	1.1	0.9,1.2	2911	−0.1	−0.3,0.0	2450	−0.4	−0.5,−0.3	1633	−1.6	−1.9,−1.4
	High	1453	1.1	1.0,1.3	1564	−0.3	−0.4,−0.1	1417	−0.4	−0.5,−0.4	984	−1.2	−1.5,−0.9
	*P* value	0.319			0.002			0.338			0.125		

*Abbreviations*: *BMI* body mass index, *WC* waist circumference, *CI* confidence interval, *SR* self-reported, *DM* directly measured

Overweight or obese respondents were more likely to overestimate their height and underestimate their weight, BMI, and WC compared to normal and underweight respondents (*P* < 0.001), as did participants with elevated WC compared with those with normal WC, all *P* < 0.001 (Table 2).

**Table 2:** The difference between self-reported and measured (SR-DM) height, weight, BMI, and WC by BMI categories and WC categories in Jiangsu, China

		Height (cm)			Weight (kg)			BMI (kg/m^2^)			WC (cm)		
		n	Mean	95 % CI	n	Mean	95 % CI	n	Mean	95 % CI	n	Mean	95 % CI
Measured BMI categories	Underweight	128	0.4	−0.1,0.9	137	2.0	0.8,3.2	118	0.5	−0.1,1.0	72	1.0	0.2,1.8
	Normal	2247	0.9	0.8,1.0	2536	0.4	0.2,0.6	2156	−0.1	−0.2,0.0	1380	−0.8	−1.0,−0.5
	Overweight	1931	1.2	1.1,1.4	2301	−0.2	−0.4,0.0	1860	−0.5	−0.6,−0.4	1292	−2.0	−2.3,−1.7
	Obesity	731	1.7	1.3,2.1	893	−1.3	−1.6,−1.0	695	−1.2	−1.4,−1.0	525	−2.6	−3.1,−2.1
	*P* value	< 0.001			< 0.001			< 0.001			< 0.001		
Measured WC	Normal	3306	1.1	0.9,1.3	3771	0.3	0.1,0.5	2615	−0.2	−0.3,−0.1	2084	−0.2	−0.4,−0.0
	Elevated	1731	1.2	1.0,1.4	2095	−0.4	−0.6,−0.2	2214	−0.6	−0.7,−0.5	1185	−2.8	−3.0,−2.6
	*P* value	0.137			< 0.001			< 0.001			< 0.001		

*Abbreviations BMI* body mass index, *WC* waist circumference, *CI* confidence interval, *SR* self-reported, *DM* directly measured

Respondents who were not daily smokers, had intakes of fruits/vegetables ≥400 g/d, or those with sufficient physical activity level were more likely to overreport their height (*P* < 0.001). Similarly, respondents who were not daily smokers, not alcohol-drinking, had less meat intakes or more fruit/vegetable intakes were more likely to underreport their WC, all *P* < 0.05. Behavioral factors were not significantly associated with the extent of bias of self-reported weight or BMI, except that smoking was associated with greater accuracy of self-reported height and weight (*P* <0.001) and meat intake ≥100 g/d with under estimation of weight (*P* = 0.033) (Table 3).

**Table 3:** The difference between self-reported and measured (SR-DM) height, weight, BMI, and WC by lifestyle factors in Jiangsu, China

		Height (cm)			Weight (kg)			BMI (kg/m^2^)			WC (cm)		
		n	Mean	95 % CI	n	Mean	95 % CI	n	Mean	95 % CI	n	Mean	95 % CI
Current daily smoking	No	3870	1.2	1.1,1.3	4557	−0.1	−0.3,0.0	3709	−0.5	−0.5,−0.4	2613	−1.7	−1.9,−1.5
	Yes	1167	1.0	0.8,1.1	1310	0.1	−0.1,0.3	1120	−0.2	−0.3,−0.1	656	−0.7	−1.1,−0.2
	*P* value	0.026			0.061			< 0.001			< 0.001		
Drinking alcohol within 30 days	No	3656	1.2	1.1,1.3	4294	−0.1	−0.2,0.1	3502	−0.4	−0.5,−0.3	2471	−1.6	−1.8,−1.4
	Yes	1381	1.1	0.9,1.2	1573	−0.1	−0.3,0.1	1327	−0.4	−0.5,−0.3	798	−1.1	−1.5,−0.7
	*P* value	0.347			0.833			0.945			0.01		
Meat intake	<100 g/d	4248	1.2	1.1,1.2	4997	0.0	−0.1,0.1	4072	−0.4	−0.4,−0.3	2800	−1.5	−1.7,−1.3
	≥100 g/d	715	1.0	0.7,1.2	779	−0.4	−0.6,−0.1	687	−0.5	−0.6,−0.3	419	−1.0	−1.5,−0.5
	*P* value	0.188			0.033			0.228			0.048		
Fruits/vegetables intake	≥400 g/d	2774	1.3	1.2,1.4	3050	−0.1	−0.2,0.1	2663	−0.4	−0.5,−0.4	1810	−1.3	−1.6,−1.1
	<400 g/d	2235	0.9	0.8,1.1	2786	−0.1	−0.2,0.1	2138	−0.4	−0.4,−0.3	1442	−1.7	−2.0,−1.5
	*P* value	< 0.001			0.991			0.114			0.026		
Physical activity level	Insufficient	1008	0.9	0.6,1.1	1110	−0.1	−0.4,0.2	967	−0.4	−0.5,−0.3	625	−1.3	−1.7,−0.9
	Moderate	1392	1.0	0.9,1.2	1548	−0.2	−0.4,0.0	1344	−0.4	−0.5,−0.3	883	−1.4	−1.7,−1.1
	Sufficient	2637	1.3	1.2,1.4	3209	0.0	−0.1,0.2	2518	−0.4	−0.5,−0.3	1761	−1.6	−1.9,−1.4
	*P* value	< 0.001			0.18			0.742			0.393		

*Abbreviations*: *BMI* body mass index, *WC* waist circumference, *CI* confidence interval, *SR* self-reported, *DM* directly measured

**Fig. 2:**
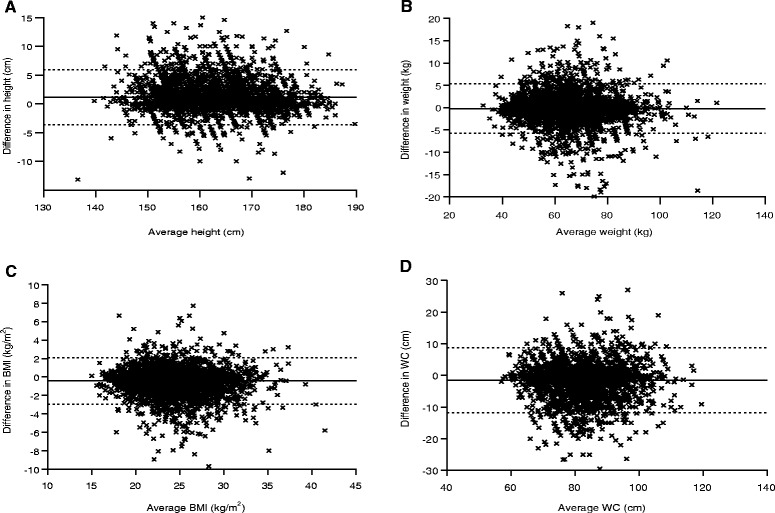
Bland-Altman plots of the difference (self-reported minus measured) against the average ((self-reported + measured)/2) of height (**a**), weight (**b**), BMI (**c**) and WC (**d**). Solid line represents the mean difference and dotted line represents the 95 % limits of agreement. BMI: body mass index; WC: waist circumference

The majority of differing values between self-reported and measured data fell within the limits of agreement, indicating a fairly good level of agreement between self-reported and measured data (Fig. 2). Bland-Altman plots demonstrated an approximately normal distribution of error in self-reported height but an uneven distribution of error in self-reported weight, BMI, and WC. The plots indicated that higher values of average weight ((self-reported + measured)/2) were related to a greater variance of bias in weight. Participants with BMI around 25 kg/m^2^ or with WC between 80 cm and 100 cm had much greater variance in the bias of self-reported BMI or WC.

Of respondents whose directly measured height and weight values categorized them as obese according to the Chinese standard, 25.5 % were misclassified (i.e., classified as overweight or normal weight) based on self-reported height and weight. A greater proportion of misclassification of weight status was observed among women than among men (35.8 % vs. 11.6 %). Based on self-reported WC, 16.3 % of women and 2.6 % of men (8.7 % in all) with elevated WC (according to the IDF standard for Chinese) on direct measurement were misclassified as normal. Such proportions decreased to 21.0 % in the obesity misclassification and conversely increased to 10.6 % in the elevated WC when categorizing by the World Health Organization standard and the IDF standard for Americans, respectively (Table 4).

**Table 4:** Errors in the classification of BMI categories and WC categories based on self-reported anthropometrics in Jiangsu, China (%)

Categories	SR			DM			Absolute Error			Relative Error		
	Men	Women	All	Men	Women	All	Men	Women	All	Men	Women	All
BMI categories (kg/m^2^, according to the Chinese standard)												
< 18.5	2.9	3.7	3.3	2.3	2.4	2.3	0.6	1.3	1.0	27.0	56.1	42.6
18.5–23.9	45.9	51.4	48.8	43.5	43.3	43.4	2.4	8.1	5.4	5.6	18.7	12.5
24.0–27.9	38.6	34.6	36.5	40.0	38.3	39.1	−1.4	−3.7	−2.6	−3.5	−9.7	−6.5
≥ 28.0	12.5	10.3	11.4	14.2	16.0	15.2	−1.6	−5.7	−3.9	−11.6	−35.8	−25.5
WC categories (cm, according to the IDF standard for Chinese)												
< 90 of men/ <80 of women	64.4	51.3	56.4	66.1	41.8	52.3	−1.7	9.5	4.2	−2.6	22.6	8.0
≥ 90 of men/ ≥80 of women	35.6	48.7	43.6	33.9	58.2	47.7	1.7	−9.5	−4.2	5.1	−16.3	−8.7
BMI categories (kg/m^2^, according to the WHO standard)												
< 18.5	3.0	5.2	4.0	2.5	4.7	3.6	0.5	0.4	0.4	20.5	9.5	11.6
18.5–24.9	61.6	69.9	65.5	57.3	60.4	58.8	4.3	9.5	6.7	7.6	15.7	11.4
25.0–29.9	29.4	21.3	25.6	33.7	29.1	31.4	−4.3	−7.8	−5.8	−12.7	−26.8	−18.5
≥ 30.0	6.0	3.7	4.9	6.6	5.8	6.2	−0.6	−2.1	−1.3	−9.0	−36.7	−21.0
WC categories (cm, according to the IDF standard for Americans)												
< 102 of men/ <88 of women	95.7	82.4	88.6	95.2	79.3	87.2	0.4	3.1	1.4	0.5	3.9	1.6
≥ 102 of men/ ≥88 of women	4.3	17.6	11.4	4.8	20.7	12.8	−0.4	−3.1	−1.4	−9.2	−15.0	−10.6

*Abbreviations*: *BMI* body mass index, *WC* waist circumference, *SR* self-reported, *DM* directly measured; *Absolute error* = SR-DM, *Relative error* = Absolute error/DM*100, *WHO* World Health Organization, *IDF* International Diabetes Federation

The results of hierarchical regression analysis are summarized in Table 5. Age group, BMI categories, and elevated WC were strong predictors for the bias in self-reported height. Intake of fruits/vegetables ≥400 g/d significantly contributed to the variance of difference between self-reported and measured height (*B* = -0.01, *P* < 0.05). Current daily smoking strongly predicted reduced bias in BMI based on self-reported height and weight (*B* = 0.02, *P* < 0.05). There were no behavioral factors statistically predictive of the bias in self-reported weight or WC (both *P* for R^2^ change of model 3 in self-reported weight and WC >0.05). Age group, location, education, income, and BMI categories were found to be associated with the error in self-reported weight. Sex, age group, location, and elevated WC were associated with that of WC.

**Table 5:** Summary of hierarchical regression models for the difference between self-reported and measured height, weight, BMI, and WC in Jiangsu, China (*B*-value)

	Height			Weight			BMI			WC		
	Model1	Model2	Model3	Model1	Model2	Model3	Model1	Model2	Model3	Model1	Model2	Model3
Gender	0.17	0.31*	0.18	−0.02	−0.04	−0.01	−0.08	−0.15*	−0.10	−0.97*	−0.58*	−0.30
Age group	0.30*	0.31*	0.30*	0.14*	0.17*	0.15*	−0.05*	−0.04	−0.04*	0.17*	0.33*	0.33*
Location	−0.09	−0.05	−0.05	0.42*	0.42*	0.44*	0.17*	0.13	0.14*	−0.81*	−0.74*	−0.70*
Education	−0.10	−0.09	−0.09	−0.15	−0.23*	−0.22*	−0.01	−0.04	−0.03	0.28	0.10	0.08
Household income level	−0.06	−0.07	−0.07	−0.20*	−0.19*	−0.19*	−0.05	−0.05	−0.05	0.03	0.01	0.00
Measured BMI categories		0.53*	0.51*		−0.83*	−0.83*		−0.56*	−0.56*		−0.18	−0.17
Elevated WC		−0.01*	−0.01*		0.00	0.00		0.00	0.00		−0.02*	−0.02*
Current daily smoking			0.00			0.00			0.02*			0.00
Drinking alcohol within 30 days			0.00			0.00			0.00			0.00
Meat intake ≥ 100 g/d			0.00			0.00			0.00			0.00
Fruits/vegetables intake < 400 g/d			−0.01*			0.00			0.00			0.00
Physical activity level			0.11			0.04			0.04			−0.03
R^2^	0.022	0.033	0.036	0.007	0.028	0.029	0.004	0.045	0.048	0.016	0.071	0.073
*F* for R^2^ Change	22.418*	26.926*	3.748*	8.472*	61.921*	1.279	3.817*	101.945*	3.272*	10.428*	95.341*	1.185

*Abbreviations*: *BMI* body mass index, *WC* waist circumference

*P < 0.05

## Discussion

This study is the first to evaluate the accuracy of self-reported height, weight, and WC among general adult Chinese, and it systematically examines the association between the bias in self-reported anthropometrics and demographic and behavioral factors. The analysis will potentially enrich the literature of accuracy of self-reported data as the Chinese traditionally believe that fatness is a symbol of abundance rather that a health problem [17], which is culturally different to the Western populations previously studied. Meanwhile, understanding the accuracy of self-reported anthropometrics in this population is important because the prevalence of obesity has increased considerably in the past two decades, and obesity has become one of the major public health problems [15–17].

Consistent with previous studies from other countries [2, 26], we found that self-reported and measured data of Chinese adults were highly correlated and had fairly good agreement with each other. However, self-reported height was overestimated whilst self-reported weight, BMI, and WC were all significantly underestimated, which was consistent with most existing studies [5, 8, 13, 14]. Notably, the average difference between self-reported and measured height, weight, and BMI were lower than previously reported studies performed in Western countries [2, 5, 6, 10]. This could be partly explained by the aforementioned social desirability among the Chinese that overweight or obesity is a sign of abundance rather than a health problem [17]. With the increased attention being given to overweight and obesity as major public health issues in recent years in China, people are undoubtedly becoming more sensitized to these issues. Therefore, it is possible that the self-reporting of anthropometric data in this study population could be more accurate than in previous studies.

Sex has been considered as one of the most important determinants for the accuracy of self-reported height, weight, and BMI, with more studies supporting that women were more likely to underestimate their BMI than men [3, 5, 6, 27]. However, we have observed no significant difference in the bias of self-reported height, weight, or BMI between men and women in this study population. The relatively higher prevalence of overweight or obesity among men than women in China (28.5 % vs. 24.5 %) [15, 28] may be one of the main reasons for this, because overweight or obese people were proven to be more likely to have a greater extent of bias in self-reported data [9, 29, 30]. Another factor that may affect the sex difference in this study is the preferred units in which participants give their self-reported weight. While Chinese men tend to report weight in metric units (kg), women are more likely to have reported weight in Chinese traditional units (Jin, where 1 Jin = 0.5 kg), which may be subject to less rounding bias than if metric units were used [31].

Although self-reported data were more accurate among Chinese adults compared to that of adults from Western countries, the proportion of respondents misclassified as non-obese using self-reported data was unexpectedly high (25.5 % for all). Dekkers et al. also observed a relative error of obesity prevalence based on self-reported height and weight that was over one-fifth (33.7 % of measured vs. 26.8 % of self-reported) [2]. After changing the cut-off point standards of BMI categories and WC categories, the proportion of misclassification also changed. In other words, the reliability of estimating the prevalence of obesity according to self-reported measures not only depends on the absolute accuracy of self-reported data, but also depends on the BMI and WC distribution, as well as the cut-off point standard of obesity (overall or abdominal) in a certain population. The Bland-Altman plots demonstrated that participants with average BMI values close to the cut-off points of overweight or with average WC values close to the cut-off point of elevated WC had much greater variance of bias. This might be the reason for the high obesity misclassification rate in this research population.

Although considerable skepticism has been expressed by researchers about the accuracy and the validity of self-reported data [2, 6], for reasons of cost and practicality, they are still often used in surveillance and routine clinical assessment of obesity to calculate BMI [6, 9, 14]. To minimize biases resulting from self-reported estimates, some researchers have attempted to identify correction factors or formulas that could be applied to help minimize such bias [6, 11]. Our finding makes the feasibility of such adjustment further complicated. Researchers should be cautious of the possibility that such adjustment might lead to greater biases in estimates of BMI-related morbidity and mortality risk because, on the one hand, the BMI distribution of a population is dynamic rather than fixed, and on the other hand, even if the correcting coefficients were established for a population, it could be inappropriate to apply this to other populations due to their difference in the cut-off point standard of obesity. Meanwhile, it should not be forgotten that the proportion of misclassification of BMI categories based on self-reported data was rather high. Overall, based on the findings of this analysis as well as the results of previous studies [2, 3, 6], the self-reported anthropometric data could be biased and misleading.

The bias of self-reported WC in this study (underestimated by −1.5 cm) was in the middle of the range compared to previous studies [2, 10]. The misclassification rate of abdominal obesity was relatively lower than that of overall obesity (both underreported, 8.7 % vs. 25.5 %). Nonetheless, this does not mean that self-reported WC is better than BMI calculated from self-reported height and weight in predicting obesity, for there was much greater discrepancy and bigger fluctuation in the self-reported WC data than in BMI based on self-reported height and weight.

We observed that participants with socially perceived “healthy lifestyles,” such as not smoking or not drinking alcohol, eating less meat, consuming more fruits/vegetables, and being physically active, were more likely to overreport their height but underreport their WC. Smoking was found to be associated with more accurate BMI based on self-reported height and weight, which concurs with the findings of former studies [2, 12]. More intake of fruits/vegetables was further found to be significantly associated with greater extent of bias in self-reported height by multifactorial analysis in this study. It appears that Chinese adults with behaviors which are “aligning with social desirability” are more likely to overestimate their height and underestimate their weight and WC. Some researchers have argued that the pressures of social desirability in a face-to-face interview, as performed in the present study, may have resulted in greater bias in self-reported measures [5, 9], which could partly explain our findings on the association of certain behaviors and the accuracy of self-reported data.

By randomly sampling from a general community population, strictly adhering to a standard protocol of anthropometric measurements, relatively large sample size, together with no time lapse between self-reporting and measuring, this study provides rather reliable evidence in the accuracy of self-reported data and its determinants as well. However, we should keep in mind that the disadvantaged residents in the research population, such as the elderly, those living in rural areas, and those with low education or low income, have relatively higher unawareness rate of their anthropometrics, limiting our confidence in the findings. Moreover, it should not be forgotten that respondents of this survey did know in advance that their height, weight, and WC would be measured, possibly resulting in a bias towards more accurate reporting.

## Conclusions

In a general adult Chinese population, height was overreported whilst weight, BMI, and WC were underreported, and such bias could be affected by a multitude of factors, including demographic characteristics (including sex, age, location, and education), weight status, and behavioral factors like smoking and fruit/vegetable intake. Moreover, the obesity misclassification rate based on self-reported measures was high in the general population, and such rates were associated with the distribution of anthropometrics and the cut-off point standard of obesity, which could be different from population to population. Given the above findings, we suggest for future studies that: if anthropometric measurements are not key study variables, rather than relying on self-reported data, they should be excluded to reduce the cost of data collection and to prevent deducing an inaccurate estimation, and if anthropometric measurements are crucial variables, they should be directly measured. Though no measurement procedure is without error, we think that such error for direct measurements could be better controlled in analysis.

## Abbreviations

BMI, body mass index; CI, confidence interval; DM, directly measured; DSP, disease surveillance point; SR, self-reported; WC, waist circumference
